# Synthesis and Spectroscopic Characterization of Thienopyrazine-Based Fluorophores for Application in Luminescent Solar Concentrators (LSCs)

**DOI:** 10.3390/molecules26185428

**Published:** 2021-09-07

**Authors:** Xheila Yzeiri, Massimo Calamante, Alessio Dessì, Daniele Franchi, Andrea Pucci, Francesco Ventura, Gianna Reginato, Lorenzo Zani, Alessandro Mordini

**Affiliations:** 1 Department of Chemistry “Ugo Schiff”, University of Florence, Via della Lastruccia 13, 50019 Sesto Fiorentino, Italy; xheila.yzeiri@stud.unifi.it (X.Y.); alessandro.mordini@unifi.it (A.M.); 2 CNR-Institute of Chemistry of Organometallic Compounds (CNR-ICCOM), Via Madonna del Piano 10, 50019 Sesto Fiorentino, Italy; a.dessi@iccom.cnr.it (A.D.); daniele.franchi@iccom.cnr.it (D.F.); lorenzo.zani@iccom.cnr.it (L.Z.); 3 Department of Chemistry and Industrial Chemistry, University of Pisa, Via G. Moruzzi 13, 56124 Pisa, Italy; andrea.pucci@unipi.it (A.P.); f.ventura6@studenti.unipi.it (F.V.)

**Keywords:** organic fluorophores, NIR emission, thienopyrazine, luminescent solar concentrators

## Abstract

Organic fluorophores have found broad application as emitters in luminescent solar concentrators (LSCs) for silicon photovoltaics. In particular, the preparation of organic conjugated systems with intense light-harvesting ability, emissions in the deep-red and NIR regions, and large Stokes shift values represent a very challenging undertaking. Here, we report a simple and easy way to prepare three symmetrical donor–acceptor–donor (DAD) organic-emitting materials based on a thienopyrazine core. The central core in the three dyes was modified with the introduction of aromatic substituents, aiming to affect their optical properties. The fluorophores were characterized by spectroscopic studies. In all cases, visible-NIR emissions with large Stokes shifts were found, highlighting these molecules as promising materials for the application in LSCs.

## 1. Introduction

The investigation of efficient systems to replace fossil fuels with more sustainable energy sources has been one of the most challenging research fields of recent years. In particular, the development of new technologies for converting sunlight into other forms of energy, such as electricity, represents a key issue as solar light is the most abundant renewable source and is well-distributed all over the planet [1].

Currently, the main technology used to exploit solar energy consists of silicon photovoltaic (PV) panels, but several other emerging devices have been proposed [2,3,4,5]. The main goal for their development is that of overcoming some of the drawbacks connected with the use of traditional PVs, such as the need for direct light irradiation, limited performance under low light, difficult urban integration, and poor dispersion of excess heat due to unconverted energy. One of the proposed solutions is based on luminescent solar concentrators (LSCs), which are exploited as an alternative approach to lowering the costs of PVs and facilitating the integration of solar-harvesting devices into buildings [6].

**Figure 1:**
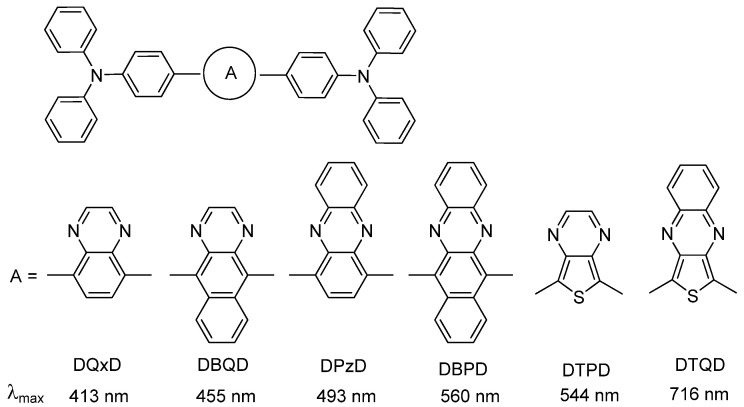
Structure of benzopyrazine- and thienopyrazine-based chromophores with triphenylamine donors.

LSCs rely on a technology studied since the 1970s [7,8] and, generally, consist of transparent polymer sheets doped with luminescent species. Incident sunlight is absorbed by the luminescent compounds and emitted at longer wavelengths. Thanks to the different refractive indexes of air and the dispersing matrix, the emitted radiation is then mostly concentrated, via total internal reflection, at the edges of the panel, where traditional silicon solar cells are placed. Commonly used fluorescent compounds can be quantum dots, perovskites, rare-earth complexes, and organic molecules [9], possibly characterized by high fluorescence quantum yields, large Stokes shifts, and a suitable match between the emission wavelengths and the electronic band-gap of the PV cell used. Among them, small organic molecules with a donor–acceptor–donor (DAD) type structure appear particularly interesting because their band-gap levels and other related properties can be readily tuned by introducing a variety of donor and acceptor moieties. In particular, benzo- and thienopyrazines, such as those shown in Figure 1, can be promising fluorophores for LSC applications as they have been found to have strong intramolecular charge transfer (ICT) transitions [10] due to the electron-withdrawing properties of the pyrazine ring coupled with the electron-donating properties of the flanking triphenylamine groups. 

In addition, it has been shown that by extending the conjugation of the pyrazine acceptor in an orthogonal direction to the DAD backbone, the ICT transitions are dramatically strengthened, lowering the band-gap and resulting in a bathochromic shift of the emission of the corresponding compounds. Such property could be highly beneficial in view of their employment in LSCs, thanks to the improved spectral matching with the absorption of *c*-Si photovoltaic cells, extending until the near-IR (NIR) region.

**Figure 2:**
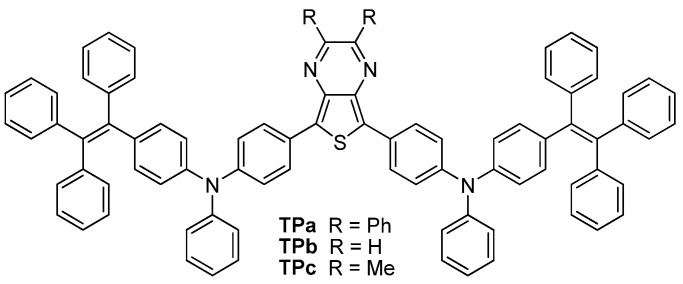
Structure of thienopyrazine-based AIE dyes.

Very recently, we reported some novel thienopyrazine-based organic dyes bearing tetraphenylethylene (TPE) moieties onto the triarylamine donor groups, which also revealed intense light-harvesting ability and emissions in the deep-red and NIR regions, with large Stokes shift values (Figure 2). Remarkably, the dyes exhibited aggregation-induced emission (AIE) properties, enhancing their emissive ability in the aggregate state [11].

**Figure 3:**
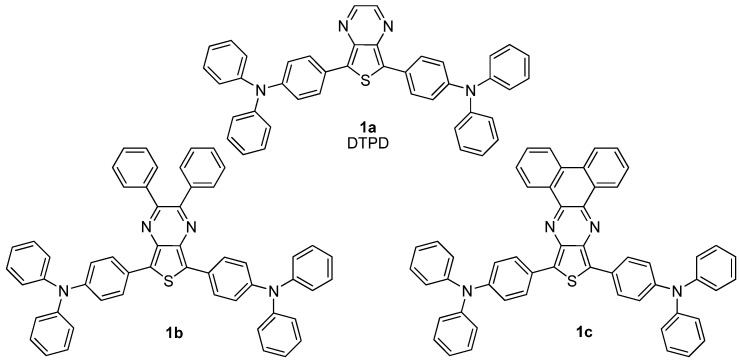
Structures of thienopyrazine-based dyes prepared in this work.

Despite the above-mentioned studies, to the best of our knowledge, no thienopyrazine-based molecule has yet been applied as an LSC emitter. In addition, the employment of compounds with emission maxima above 650 nm has been rarely reported [9]. To better understand the structure-property relationships of thienopyrazine-based emitters and explore their potential employment as NIR-emitting fluorophores for LSC application, we decided to prepare a new small family of dyes bearing such a heterocyclic core and accurately study their spectroscopic properties. Starting from known compound **1a** (DTPD), we chose to maintain the triarylamine moiety as the donor group and to change the acceptor core by extending its conjugation, introducing aromatic substituents. The structures we designed are reported in Figure 3.

Compounds **1a**–**c** were prepared using an efficient and general procedure. They were fully characterized, and, for all the dyes, spectroscopic and solvent-dependent photoluminescence studies were carried out. In all cases, emissions extending to the IR and NIR regions and large Stokes shifts were found, highlighting these molecules as promising materials for application as emitters in LSCs. One of the three dyes was dispersed in a PMMA matrix, the corresponding LSC devices were prepared, and their optical efficiencies were measured and compared with a classical reference dye, BASF Lumogen Red 305 (LR 305).

## 2. Results

### 2.1. Synthesis of Dyes

The first point to address was to find a simple and general way, possibly suitable to large-scale manufacturing, to prepare the three fluorophores, **1a**–**c**.

**Scheme 1:**
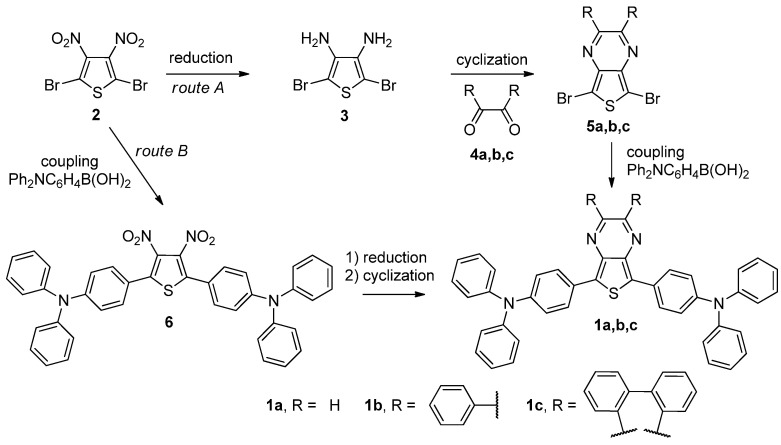
Possible synthetic routes to fluorophores **1a**–**c**.

**Scheme 2:**
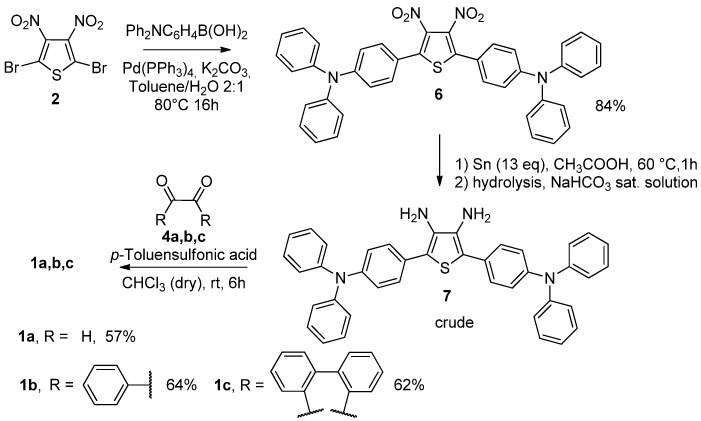
Synthetic procedure of compounds **1a**–**c**.

We identified two different synthetic approaches, which are reported in Scheme 1 (route A and route B). In both cases, 2,5-dibromo-3,4-dinitrothiophene (**2**), prepared using a previously reported procedure [12], was used as starting material. However, following route A, we observed poor conversion yields in both the reduction of compound **2** to 2,5-dibromo-3,4-diaminothiophene (**3**) and the subsequent ring-closure reaction. Accordingly, we found route B to be more general, leading to the best overall yields, and optimized the synthesis, as shown in Scheme 2. Following an already-reported procedure [10], advanced intermediate **6** was prepared in high yield through Suzuki-Miyaura coupling between (4-(diphenylamino)phenyl)boronic acid and 2,5-dibromo-3,4-dinitrothiophene (**2**). The following step was more challenging, as after the catalytic hydrogenation of the two nitro groups using Pd/C [13], a complex mixture of 3-amino-4-nitro- and 3,4 diamino-derivatives was recovered, together with several by-products.

No better result was obtained using metallic iron in acetic acid as the reducing agent [14,15]. Nevertheless, a good yield of diamine (**7**) was finally obtained using powdered tin as a reductant in acetic acid at 60 °C. Under these conditions, satisfactory yields of the desired diamine were found in the crude mixture, following hydrolysis with a basic bicarbonate solution. Unfortunately, all the attempts made to purify intermediate **7** were unsuccessful, making it necessary to use the crude mixture directly for the subsequent cyclization step. Conversion to the pyrazine ring was performed in the presence of the suitable 1,2-dicarbonyl compound, using dry CHCl_3_ as a solvent and *p*-toluenesulfonic acid as a catalyst at room temperature. Under these conditions, condensation with glyoxal (**4a**), benzil (**4b**), and phenanthrene-9,10-dione (**4c**) gave the corresponding thienopyrazines **1a**–**c** in 57%, 64%, and 62% overall yield, respectively (Scheme 2). After purification, the resulting compounds were characterized by ^1^H- and ^13^C-NMR and HRMS spectroscopies (see Supplementary Materials), the results of which were consistent with the proposed structures.

### 2.2. Photophysical Properties

**Figure 4:**
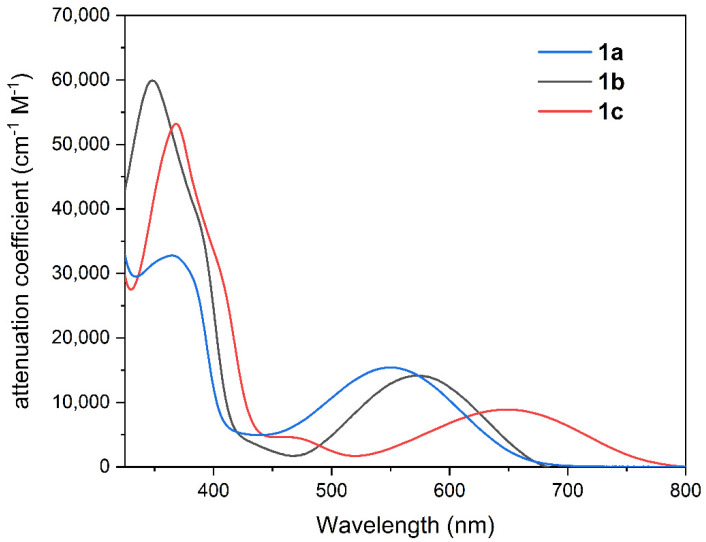
UV-vis absorption spectra of the three compounds in toluene (conc. 0.01 mM). **1a**: blue line; **1b**: black line; **1c**: red line.

The synthesized compounds **1a**–**c** were characterized after dissolution in different solvents through UV-vis and photoluminescence (PL) spectroscopy measurements. First, experiments were conducted in toluene since it effectively dissolved all compounds, and its refractive index (1.496) is similar to that of PMMA (1.491), that is, the polymer matrix commonly used in LSC experiments (see below), thus providing the best reference for the following application. The UV-vis spectra of the three compounds showed two main absorption bands at different wavelengths, a more intense one in the near-UV region and another one in the visible range (Figure 4). 

The band in the near-UV region was attributed to a localized π→π* transition of the donor triphenylamine groups [16], while the lower energy band was due to the internal charge transfer (ICT) transition from the donor moiety to the acceptor thienopyrazine core [10]. Spectroscopic data, reported in Figure 4 and Table 1, clearly show that extending the conjugation of the thienopyrazine core has a pronounced bathochromic effect on the ICT transition; indeed, the unsubstituted compound **1a** features a 552 nm absorption maximum wavelength in toluene solution, while for diphenyl-substituted **1b**, the ICT band is red-shifted by 19 nm (λ_max_ = 571 nm). Finally, the highly conjugated dibenzo[*f*,*h*]thieno[3,4-*b*]quinoxaline derivative **1c** shows a 95 nm bathochromic shift (λ_max_ = 647 nm) in comparison with **1a**. As shown in Table 1, when using solvents of different polarities, none of the compounds presented a large solvatochromism, neither of the UV-centered nor the visible absorption band, which were shifted by 12 nm at most. 

**Figure 5:**
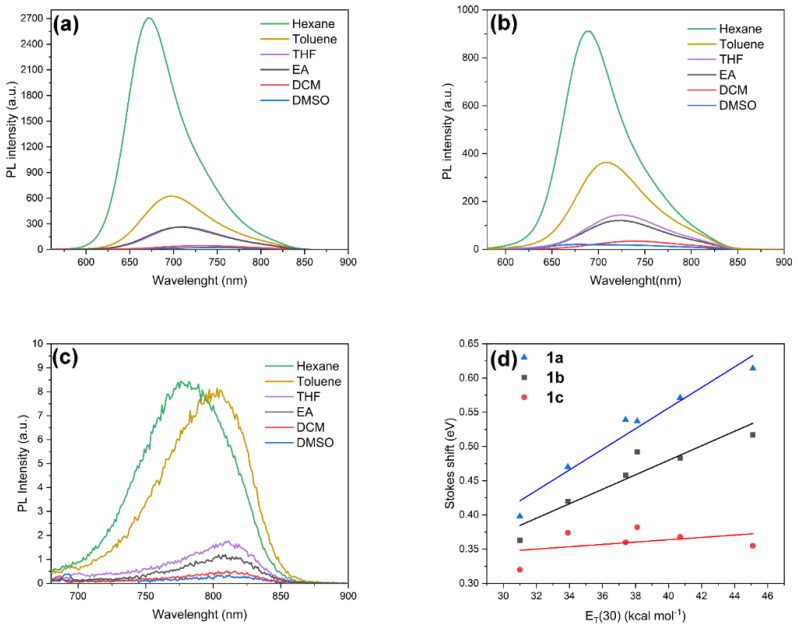
Emission band of **1a**–**c** (0.01 mM): (**a**) **1a** (λ_exc_ = 570 nm); (**b**) **1b** (λ_exc_ = 541 nm); (**c**) **1c** (λ_exc_ = 647 nm); (**d**) Stokes shift against Dimroth–Reichardt’s solvent polarity parameter E_T_(30): solid lines are fitting lines for each data set.

Next, the fluorescence emission properties of the molecules were analyzed after excitation close to the maximum absorption wavelength of the visible band region (541–647 nm, Table 1 and Figure 5). In toluene solution, **1a**,**b** presented emission bands around 700 nm, while a significantly red-shifted fluorescence was observed for **1c**, peaking in the NIR region just above 800 nm. In all cases, large Stokes shifts of 137–157 nm were observed. As it could be expected, fluorescence quantum yields were relatively low (approx. 1–4%) as a consequence of the so-called “energy gap law” [17], which states that quick non-radiative decay from low-lying excited states, favored by fast vibrational relaxation, can serve as an efficient deactivation pathway to quench NIR emission. The values found in this study were nevertheless comparable to those already observed for push–pull fluorophores emitting in the same wavelength range [18]. Unlike the rather small solvatochromism observed in the absorption spectra, all the luminophores displayed significant solvent-dependent photoluminescence properties.

This type of behavior is characteristic of transitions due to intramolecular charge transfer [10,11,19] since the electron density of the ground state S_0_ is mainly localized on the triphenylamine donor group and, due to the photoexcitation, is transferred to the electron-poor thienopyrazine core. This induces an increase in the dipole moment of the molecule in the excited state S_1_, which is, therefore, more stabilized by polar solvents, resulting in a red-shift of the emission spectra. To more accurately evaluate this behavior, the trend of the Stokes shift against Dimroth–Reichardt’s solvent polarity parameter E_T_(30), which is essentially a polarity scale based on the static dielectric constant f(D) [20,21], was assessed and is reported in Figure 5d. The greater the slope of the fitting line, the larger the difference in the dipole moments of the ground and first excited state. As it can be seen, plots for compounds **1a** and **1b** present a very similar slope; on the contrary, this is clearly lower in the case of **1c**, showing that photoexcitation causes a smaller change in the dipole moment, in agreement with better negative charge delocalization due to the larger conjugation of the dibenzo[f,h]thieno[3,4-*b*]quinoxaline core. To confirm these findings, compounds **1a**–**c** could be studied with electric-field-modulated absorption (EA) spectroscopy: indeed, by recording the variation of UV-vis absorption spectra of samples when immersed in electric fields of different strength, it is possible to determine the difference of electric dipole moments between the excited and ground states of organic compounds. This technique has already been employed in the study of donor-acceptor molecules with an application in optoelectronics [22,23]. 

In addition to the bathochromic effect of the emission band, there is a decrease in the intensity of the fluorescence emission (and, therefore, of the quantum yield) passing from non-polar solvents, such as hexane and toluene, to more polar solvents such as EA and DCM. Accordingly, the emission in DMSO (the most polar solvent among those tested) is practically zero. Again, this pattern is typical of an ICT transition, where the excited state stabilization in more polar solvents is correlated to quicker non-radiative decay compared to less polar media, in agreement with the above-mentioned energy gap law. 

**Figure 6:**
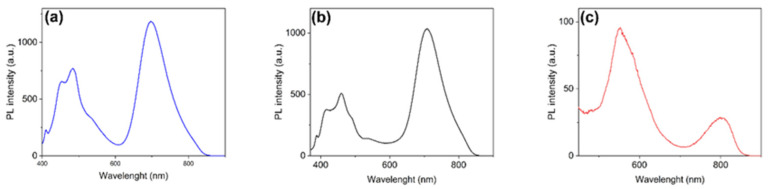
PL spectra of **1a**–**c** in toluene (0.01 mM): (**a**) **1a** (_λexc_ = 365 nm); (**b**) **1b** (λ_exc_ = 347 nm); (**c**) **1c** (λ_exc_ = 372 nm).

When the three compounds **1a**–**c** were excited in toluene at the maximum absorption wavelength in the near-UV region (347–372 nm), they presented different emission behavior. Compounds **1a**,**b** displayed a fluorescence spectrum with two emission bands (Figure 6a,b), the main of which corresponded to the peak in the near-IR region already observed in Figure 5. In other words, their behavior was substantially adherent to Kasha’s rule, stating that the fluorescence (singlet) emission of an organic compound occurs in appreciable yield only from the lowest (singlet) excited state [24].

The same, however, was not true for compound **1c**, whose main emission band was located in the visible region at around 530 nm, while the NIR band at around 800 nm was only faint (Figure 6c). A possible explanation is that the S_1_ ICT excited state for compound **1c** is much lower in energy compared to those of compounds **1a**,**b**, as demonstrated by its red-shifted absorption in the visible region (Figure 4). As a consequence, the energy difference with the S_2_ state, stemming in all cases from a localized π→π* transition centered on the triphenylamine groups (see above), would be larger in the former compound compared to the latter, slowing down the corresponding non-radiative transition and, thus, avoiding the quenching of radiative emission from the S_2_ state. 

Further studies would anyway be necessary to confirm this hypothesis, as other apparent violations of Kasha’s rule have already been traced back to the occurrence of different phenomena (e.g., tautomerism, presence of impurities) [25,26].

Thanks to the emissions in the visible and NIR regions and the large Stokes shifts showed by the fluorophores, we decided to prepare some test (50 × 50 × 3 mm^3^) LSC devices with the new emitters. Thus, the three compounds were dispersed within poly(methyl methacrylate) (PMMA), which is the most used polymer matrix for LSC devices. Disappointingly, only compound **1c** proved to be stable enough (at least for several hours under illumination) to allow full characterization of the thin films, as the samples prepared with compounds **1a**,**b** showed progressive decoloration even during room temperature storage for a few days. Such findings might be explained with the more conjugated structure of **1c** [27] compared to **1a**,**b**, which is responsible for a wider and more efficient delocalization of the electronic charge resulting from excitation and ICT transition and, consequently, for the stabilization of the excited state (as it is evident from its red-shifted emission and the solvent dependence studies; Figure 5). 

**Figure 7:**
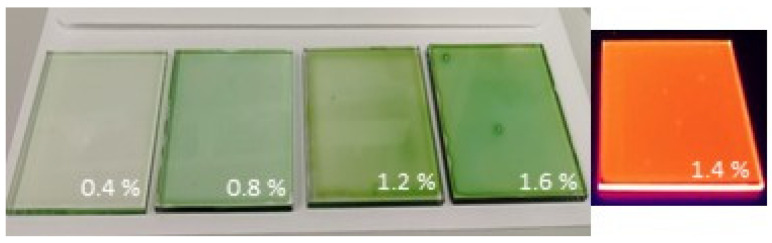
Examples of 50 × 50 × 3 mm^3^ LSC devices containing compound **1c** at different concentrations (**left**), as well as LR305 at 1.4 wt% (**right**).

Accordingly, an investigation of device properties was successfully carried out only using fluorophore **1c**. After dispersion in PMMA at different concentrations (from 0.4 to 2.0% by weight), the compound gave transparent and homogeneous films for all the investigated ranges of concentration (Figure 7).

**Figure 8:**
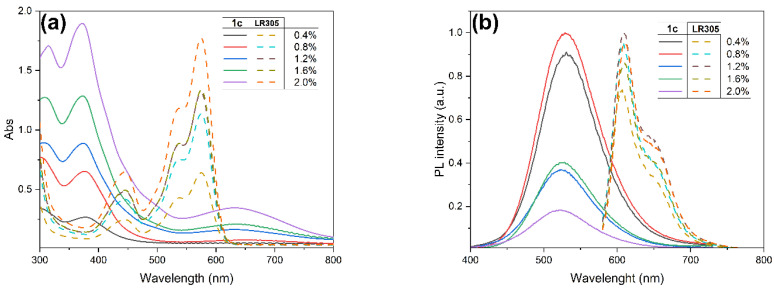
(**a**) Absorption spectra of **1c** (solid line) and **LR305** (dash line) in PMMA at different concentrations (weight %); (**b**) fluorescence emission spectra of **1c** (solid line, excitation at 372 nm) and LR305 (dash line, excitation 538 nm) in PMMA at different concentrations (weight %).

UV-vis absorption and fluorescence spectra of the films as a function of fluorophore concentration (wt%), together with fluorescence quantum yield and optical efficiencies of the corresponding LSC devices, were measured at different concentrations and are reported in Figure 8 and Table 2. The intensity of the absorption spectra grew progressively with the compound concentration, and no shape variation was observed in the curves, highlighting the good dispersion of compound **1c** in PMMA.

The emission spectra and quantum yields were evaluated by exciting the molecule at the wavelength of maximum absorption in the near-UV region (372–378 nm) due to its higher intensity compared to the ICT band: in agreement with the measurements carried out in solution, the only strong emission band observed in these conditions was that at λ_max_ ~ 520–530 nm, while the peak due to the emission from the lowest excited state was extremely weak (Figure 8b, solid lines).

By looking at the spectra of compound **1c** reported in Figure 8a,b, it can be seen that the absorption band peaking at 630 nm appears much weaker than that in the near-UV region and that the visible emission around that wavelength is only around 25% of its maximum intensity. On the other hand, it should also be noted that the intensity of fluorescence decreased when the concentration of **1c** in PMMA increased (see the quantum yield values in Table 2), and this was also accompanied by a progressive blue shift from 529 to 521 nm. Such observations lead us to think that some re-absorption of light emitted from the higher energy excited state is present in the LSC, and, therefore, tuning the concentration of the emitter in PMMA should be crucial to maximizing device efficiency. Nevertheless, the Stokes shift for this transition was still very large (>0.9 eV), constituting a favorable condition for the employment of **1c** in LSCs, effectively limiting re-absorption phenomena. On the other hand, the quantum yield collapse, observed with increasing fluorophore concentration, could also be due to the formation of aggregates, triggering the typical “aggregation–caused quenching” (ACQ) effect [28]. 

Finally, the performances of the fluorophore/polymer films as LSCs were determined according to the procedure reported in the experimental part. The power generated by a c-Si photovoltaic cell was measured upon both direct exposure to the light of an AM 1.5G solar simulator (P_SC_) and when connected to the LSC (P_LSC_). The P_LSC_/P_SC_ ratio is the concentration factor (C) linked to optical efficiency (η_opt_) through the LSC geometric factor (G), which is simply the ratio between the area of the LSC and that of the bare (masked) *c*-Si cell. The results obtained with compound **1c** were compared with those collected from the reference dye Lumogen Red 305 (**LR305**, Table 2) under the same conditions. Although the quantum yield of **1c** dispersed in PMMA was quite low (16% max) when compared to that of **LR305**, the optical efficiency of 6.81% that we observed at concentration 1.2 wt% was quite satisfactory, especially because it was accompanied by a concentration factor higher than 1, which is the minimum required condition for a light collector; this is not so commonly observed for this kind of molecule [9]. Such positive results are probably derived from the good spectral matching between light absorption by the *c*-Si PV cell and the fluorophore emission, especially when irradiated with light of different wavelengths, coupled with the large Stokes shifts that limited light re-absorption within the LSC device. Taken together, these observations promote the further exploration of the compounds featuring a dibenzo[*f*,*h*]thieno[3,4-*b*]quinoxaline heterocyclic core as potential organic emitters for LSC applications.

## 3. Materials and Methods

### 3.1. General Information

Unless otherwise stated, all reagents were purchased from commercial suppliers and used without further purification. 2,5-dibromo-3,4-dinitrothiophene (**2**) was prepared as previously reported [12]. All air-sensitive reactions were performed using Schlenk techniques. Solvents used in cross-coupling reactions were previously degassed by means of the “freeze–pump–thaw” method. Tetrahydrofuran (THF) was freshly distilled immediately before use from sodium/benzophenone. CH_2_Cl_2_, toluene, and acetonitrile were dried on a resin exchange solvent purification system. Petroleum ether, unless specified, is of the 40–70 °C boiling fraction. For all spectroscopic measurements, solvents with spectroscopy grade purity were used.

Organic phases derived from aqueous work-up were dried over Na_2_SO_4_. Reactions were monitored by TLC on SiO_2_ plates, and the detection was made using a KMnO_4_ basic solution or UV lamp. Flash column chromatography was performed using glass columns (10–50 mm wide) and SiO_2_ (230–400 mesh). ^1^H-NMR spectra were recorded at 200 or 400 MHz and ^13^C-NMR spectra at 50.0 or 100.6 MHz, respectively. Chemical shifts were referenced to the residual solvent peak (CDCl_3_, δ 7.26 ppm for ^1^H-NMR and δ 77.16 ppm for ^13^C-NMR; THF-*d*_8_ δ 3.58 and 1.72 ppm for ^1^H-NMR, δ 67.21 and 25.31 ppm for ^13^C-NMR; CD_2_Cl_2_, δ 5.32 ppm for ^1^H-NMR, δ 53.84 ppm for ^13^C-NMR). Coupling constants (*J*) are reported in Hz. ESI-MS spectra were recorded with a Thermo Fisher LCQ-Fleet Ion-Trap mass spectrometer. HR-MS measurements were performed using a Thermo Fisher LTQ Orbitrap FT-MS spectrometer (Waltham, MA, USA). FT-IR spectra were recorded with a Perkin-Elmer Spectrum UATR (Waltham, MA, USA) instrument in the range of 4000–400 cm^−1^ with a 2 cm^−1^ resolution. UV–vis spectra were recorded with a Shimadzu UV2600 series spectrometer (Kyoto, Japan), and fluorescence spectra in solution were recorded with a Jasco FP-8300 instrument (Tokyo, Japan), irradiating the sample at the wavelength corresponding to maximum absorption in the UV-vis spectrum. Fluorescence spectra on polymer films were measured at room temperature with a Horiba Jobin–Yvon Fluorolog^®^-3 spectrofluorometer (Horiba Jobin–Yvon, Horiba Italy, Rome, Italy) equipped with a 450 W Xenon arc lamp and double-grating excitation and single-grating emission monochromators.

The concentration factors and optical efficiencies of the LSCs were obtained using a solar simulator (ORIEL^®^ LCS-100 solar simulator (Newport, North Kingstown, RI, USA), AM1.5G std. filter: 69 mW/cm^2^ at 254 mm) and a calibrated PV cell (IXYS SLMD121H08L mono solar cell 86 × 14 mm: Voc = 5.04 V, Isc = 50.0 mA, FF > 70%, η = 22%) connected to a precision source/measure unit (Keysight Technologies B2900 Series) (Santa Rosa, CA, USA). The PV cell was masked with black tape to match the LSC edge (50 × 3 mm) to make stray light negligible [29]. High purity silicon was used to grease the PV cell to the LSC edge to limit flux losses. Only one edge of the waveguide was attached to the PV cell to make the wiring connections simple. The other three edges of the LSC were covered with reflective aluminum tape in agreement with the literature [30]. Optical efficiency (η_opt_) was determined from the concentration factor, i.e., the ratio between the maximum power measured for the cell over the LSC edge (P_LSC_) and that of the bare cell when exposed to the light source (P_SC_), according to the following Equation (1):

(1) $$\eta_{\mathrm{opt}}=\frac{P_{\mathrm{LSC}}}{P_{\mathrm{SC}}\times G}$$

where C is the concentration factor and G is the geometrical factor (G = 16.6), that is, the ratio between the area exposed to the solar simulator and the collecting area by the PV cell. Notably, during the P_LSC_ measurements, a white backscattering layer (ERGA TAPES S.r.l., Microcellular MCPET reflective sheet) was placed beneath the LSC, with an air gap of about 5 mm. The reported η_opt_ values were calculated as the average of three distinct measurements.

### 3.2. Synthesis

#### 3.2.1. Synthesis of 4,4′-(3,4-Dinitrothiophene-2,5-diyl)bis(*N*,*N*-diphenylaniline) (**6**)

2,5-Dibromo-3,4-dinitrothiophene (**2**) (400 mg, 1.21 mmol, 1.0 equivalent) and 4-(diphenyl amino) benzeneboronic acid (760 mg, 2.6 mmol, 2.1 equivalents) were dissolved into a toluene/H_2_O mixture (2/1 *v*/*v*, 75 mL). [Pd(PPh_3_)_4_] (130 mg, 0.12 mmol, 10 mol%) and K_2_CO_3_ (1.64 g, 12 mmol, 10 equivalents) were then added, and the reaction mixture was warmed-up to 80 °C and stirred for 16 h. After filtration on Celite^®^, the solution was extracted with CH_2_Cl_2_ (2 × 50 mL), the organic phase washed with water and brine, and then dried. After evaporation of the solvent, the crude was purified by flash column chromatography (petroleum ether/CH_2_Cl_2_, gradient 3:1 to 1:1) to give 667 mg (yield 84%) of compound (**6**) as a red solid. ^1^H-NMR (400 MHz, CDCl_3_) δ (ppm): 7.37–7.29 (m, 12H), 7.20–7.10 (m, 12H), 7.07–7.02 (m, 4H). ^13^C-NMR (100.6 MHz, CDCl_3_) δ (ppm): 150.28, 146.66, 140.47, 130.11, 129.79, 129.76, 125.90, 124.62, 120.97, 120.13. MS (ESI) (*m*/*z*): 660.0 [M]^+^.

#### 3.2.2. General Procedure to Prepare 4,4′-Substituted Thieno[3,4-*b*]pyrazine-5,7-diyl)bis(*N*,*N*-diphenylaniline) (**1a**–**c**)

Compound (**6**) (1.0 equivalent) was dissolved into acetic acid and reacted with powdered Sn (13 equivalents). After warming up to 60 °C, the reaction mixture was stirred for 1 h, then diluted with H_2_O (10 mL) and filtered on Celite^®^. After extraction with CH_2_Cl_2_ (2 × 20 mL), the organic phase was washed with a saturated solution of NaHCO_3_ (20 mL), then with water and brine, and dried. The crude obtained after evaporation of the solvent was dissolved in dry CHCl_3_ (14 mL) and reacted with the appropriate dicarbonyl compound (1.1 equivalents) (**8**) and a catalytic amount of *p*-toluenesulfonic acid at room temperature for 6 h. The reaction mixture was washed with a saturated solution of NaHCO_3_ (20 mL), then with water and brine, and dried. The crude obtained after solvent removal was purified to give the desired compound.

#### 3.2.3. Synthesis of 4,4′-(Thieno[3,4-*b*]pyrazine-5,7-diyl)bis(*N*,*N*-diphenylaniline) (**1a**)

Compound **6** (100 mg, 0.15 mmol) was dissolved into acetic acid (14 mL) and reacted first with powdered Sn (230 mg, 1.95 mmol), then with glyoxal (**4a**) (40% solution in water) (175 mg, 0.16 mmol), in the presence of *p*-toluenesulfonic acid (5 mg). After work-up, the crude mixture was purified by flash column chromatography (petroleum ether/CH_2_Cl_2_/toluene = 2/1/1 *v*/*v*) to give 54 mg (57% yield) of compound **1a** as a dark magenta solid. ^1^H-NMR (400 MHz, THF-*d*_8_) δ (ppm): 8.46 (s, 2H), 8.18–8.11 (m, 4H), 7.30–7.25 (m, 8H), 7.15–7.11 (m, 8H), 7.11–7.08 (m, 4H), 7.06–7.01 (m, 4H). ^13^C-NMR (100.6 MHz, THF-*d*_8_) δ (ppm): 148.54, 148.49, 145.06, 140.89, 131.54, 130.20, 129.41, 128.27, 125.64, 124.19, 123.91. IR ν (cm^−1^): 3035, 2961, 2922, 2852, 1586, 1484, 1260. HRMS (ESI) *m*/*z* calculated for C_42_H_30_N_4_S 622.2191. Found 622.2202 [M·]^+^.

#### 3.2.4. Synthesis of 4,4′-(2,3-Diphenylthieno[3,4-*b*]pyrazine-5,7-diyl)bis(*N*,*N*-diphenylaniline) (**1b**)

Compound (**6**) (160 mg, 0.24 mmol) was dissolved into acetic acid (22 mL) and reacted first with powdered Sn (370 mg, 3.10 mmol), then with benzil (**4b**) (56 mg, 0.27 mmol), in the presence of *p*-toluenesulfonic acid (5 mg). After work-up, the crude was washed with a warm mixture of ethyl acetate and ethanol (2:1) to give 120 mg (64% yield) of compound **1b** as a deep purple solid. ^1^H-NMR (400 MHz, THF-*d*_8_) δ (ppm): 8.30–8.22 (m, 4H), 7.56–7.48 (m, 4H), 7.32–7.24 (m, 14H), 7.16–7.12 (m, 8H), 7.12–7.08 (m, 4H), 7.06–7.01 (m, 4H). ^13^C-NMR (100.6 MHz, THF-*d*_8_) δ (ppm): 153.22, 148.52, 148.47, 140.69, 139.40, 130.89, 130.71, 130.24, 129.44, 129.22, 128.78, 128.51, 125.75, 124.23, 123.83. IR ν (cm^−1^): 3061, 3034, 2962, 1588, 1482, 1327, 1274. HRMS (ESI) *m*/*z* calculated for C_54_H_38_N_4_S: 774.9713. Found: 774.9721 [M·]^+^.

#### 3.2.5. Synthesis of 4,4′-(Dibenzo[*f,h*]thieno[3,4-*b*]quinoxaline-10,12-diyl)bis(*N*,*N*-diphenylaniline) (**1c**)

Compound (**6**) (100 mg, 0.15 mmol) was dissolved into acetic acid (14 mL) and reacted first with powdered Sn (230 mg, 1.95 mmol), then with phenanthrene-9,10-dione (**4c**) (35 mg, 0.16 mmol), in the presence of *p*-toluenesulfonic acid (5 mg). After work-up, the crude was washed with warm ethyl acetate to give 72 mg (62% yield) of compound **1c** as a green solid. ^1^H-NMR (400 MHz, THF-*d*_8_) δ (ppm): 9.08 (d, *J* = 7.9 Hz, 2H), 8.49 (d, *J* = 8.0 Hz, 2H), 8.39 (d, *J* = 8.6 Hz, 4H), 7.68 (t, *J* = 7.8 Hz, 2H), 7.59 (t, *J* = 7.8 Hz, 2H), 7.37–7.28 (m, 8H), 7.24–7.16 (m, 12H), 7.09–7.04 (m, 4H). ^13^C-NMR (100.6 MHz, THF-*d*_8_) δ (ppm): 149.90, 148.60, 148.46, 143.04, 140.44, 133.55, 131.84, 131.48, 130.53, 130.28, 129.31, 128.93, 127.22, 127.10, 125.79, 124.24, 124.07. IR ν (cm^−1^): 3063, 3034, 2962, 2924, 2853, 1589, 1484, 1328, 1259. HRMS (ESI) *m*/*z* calculated for C_54_H_38_N_4_S: 772.2661. Found: 772.2658 [M·]^+^.

## Figures and Tables

**Table 1:** Optical properties of luminophores **1a**–**c** in different solvents.

Compound	Solvent ^a^	λ^1^_abs_ (nm)	ε^1^ (cm^−1^ M^−1^)	λ^2^_abs_ (nm)	ε^2^ (cm^−1^ M^−1^)	λ^2^_emi_ (nm)	Stokes Shift (nm [eV])	Φ_f_ (%)
**1a**	hexane	365	30,100	552	14,700	671	119 [0.40]	6
	toluene	365	57,400	552	15,000	698	146 [0.47]	2.6
	THF	365	33,600	542	15,900	709	167 [0.54]	<1
	EA	362	34,200	540	15,700	705	165 [0.54]	<1
	DCM	361	37,800	547	15,400	731	184 [0.57]	<1
	DMSO	365	27,600	540	12,600	737	198 [0.61]	<1
**1b**	hexane	343	33,400	572	8400	687	115 [0.36]	3
	toluene	348	59,000	571	15,100	708	137 [0.42]	<1
	THF	347	58,000	570	15,000	722	152 [0.46]	<1
	EA	345	36,700	563	9100	725	162 [0.49]	<1
	DCM	347	58,100	572	15,400	736	164 [0.48]	<1
	DMSO	350	22,700	566	5500	741	175 [0.52]	<1
**1c**	hexane	364	13,000	649	2000	780	131 [0.32]	4
	toluene	368	55,200	647	9500	804	157 [0.37]	4
	THF	367	48,000	655	7600	809	154 [0.36]	<1
	EA	365	26,100	648	4000	810	162 [0.38]	<1
	DCM	368	55,200	653	8600	810	157 [0.37]	<1
	DMSO	372	32,900	660	4700	814	154 [0.36]	<1

^a^ The concentration was 0.01 mM in all solvents.

**Table 2:** Spectroscopic properties and optical efficiency of **1c** in PMMA films.

Cmpd.	Conc. (wt%)	λ_abs_ (nm)	λ_ems_ (nm) ^a^	Φ_f_ (%) ^a^	Stokes Shift (nm [eV]) ^a^	P_LSC_ (µW)	η_opt_ (%) ^b^	C
**1c**	0.4	378, 632	529	16.7	151 [0.94]	65.7	6.49 ± 0.5	1.07
	0.8	377, 632	526	16.4	149 [0.93]	60.8	6.01 ± 0.5	1.00
	1.2	372, 632	523	11.5	151 [0.96]	68.9	6.81 ± 0.5	1.13
	1.6	372, 636	523	6.9	151 [0.96]	58.3	5.76 ± 0.5	0.96
	2.0	376, 632	521	5.5	145 [0.92]	57.3	5.66 ± 0.5	0.94
**LR305**	1.4	578	613	96.0	35 [0.13]	102.2	10.1 ± 0.5	1.68

^a^ For **1c**, following excitation at the maximum wavelength of the near-UV band (372–378 nm); ^b^ calculated using Equation (1), where P_SC_ = 61 µW and G = 16.6.

## Data Availability

The data presented in this study are available on request from the corresponding author.

## Supplementary Materials

The following are available online, Figures S1–S3: Normalized emission spectra of compounds **1a**–**c** in different solvents (0.01 mM); Figure S4–S6: Absorption spectra of compounds **1a**–**c** in different solvents; Figure S7–S9: Fluorescence emission spectra of **1a**–**c** in toluene at different concentrations; Figure S10: Absorption and fluorescence emission spectra of **1c** in PMMA; Figure S11: P(V) and I(V) curves of the photovoltaic cell in the presence and absence of LSC; copies of ^1^H- and ^13^C-NMR spectra of compounds **1a**–**c**.

## Sample Availability

Samples of the compounds **1a**–**c** are available from the authors.
