# Supplementary material for: Synthesis and Spectroscopic Characterization of Thienopyrazine-Based Fluorophores for Application in Luminescent Solar Concentrators (LSCs)
Source: Molecules. 2021 Sep 7;26(18):5428. doi: 10.3390/molecules26185428 (PMC8468226; doi:10.3390/molecules26185428)

Supporting Information

Synthesis and Spectroscopic Characterization of Thienopyrazine-Based Fluorophores for Application in Luminescent Solar Concentrators (LSCs)

Xheila Yzeiri[1], Massimo Calamante[1,2*], Alessio Dessì[2], Daniele Franchi[2], Andrea Pucci[3], Francesco Ventura[3], Gianna Reginato[2,*], Lorenzo Zani[2], Alessandro Mordini[1, 2]

[1] *Department of Chemistry "Ugo Schiff", University of Florence, Via della Lastruccia 13, 50019 Sesto Fiorentino, Italy*

[2] *CNR-Institute of Chemistry of Organometallic Compounds (CNR-ICCOM), Via Madonna del Piano 10, 50019 Sesto Fiorentino, Italy*

[3] *Department of Chemistry and Industrial Chemistry University of Pisa Via A. G. Moruzzi 13, 56124 Pisa, Italy*

* *Correspondence: gianna.reginato@iccom.cnr.it (G.R.); massimo.calamante@iccom.cnr.it (M.C.)*

Table of Contents

1. Figure **S1-S2-S3** - Normalized emission spectra of compounds **1a,1b,1c** in different solvents (0.01 mM)
2. Figure **S4-S5-S6** - Absorption spectra of compounds **1a,b,c** in different solvents
3. Figure **S7-S8-S9** - Fluorescence emission spectra of **1a,b,c** in toluene at different concentrations
4. Figure **S10** - Absorption and fluorescence emission spectra of **1c** in PMMA
5. Figure **S11** - P(V) and I(V) curves of photovoltaic cell in the presence and absence of LSC
6. Copies of ^{1}H and ^{13}C NMR spectra of compounds **1a,b,c**

1. Normalized emission spectra of compounds 1a,1b,1c in different solvents (0.01 mM)

Figure S1 - Normalized emission spectra of compound **1a** in different solvents

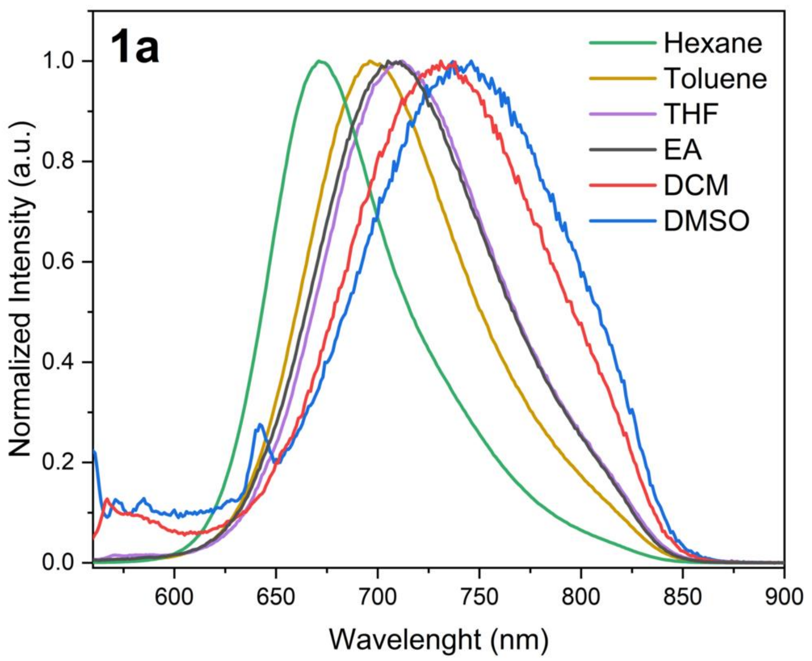

Figure S2 - Normalized emission spectra of compound **1b** in different solvents

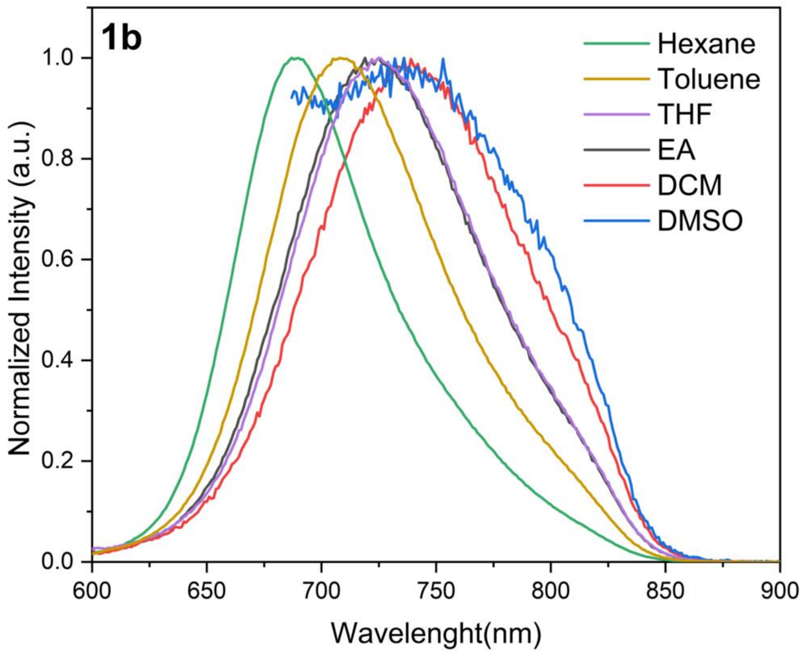

Figure S3 - Normalized emission spectra of compound **1c** in different solvents

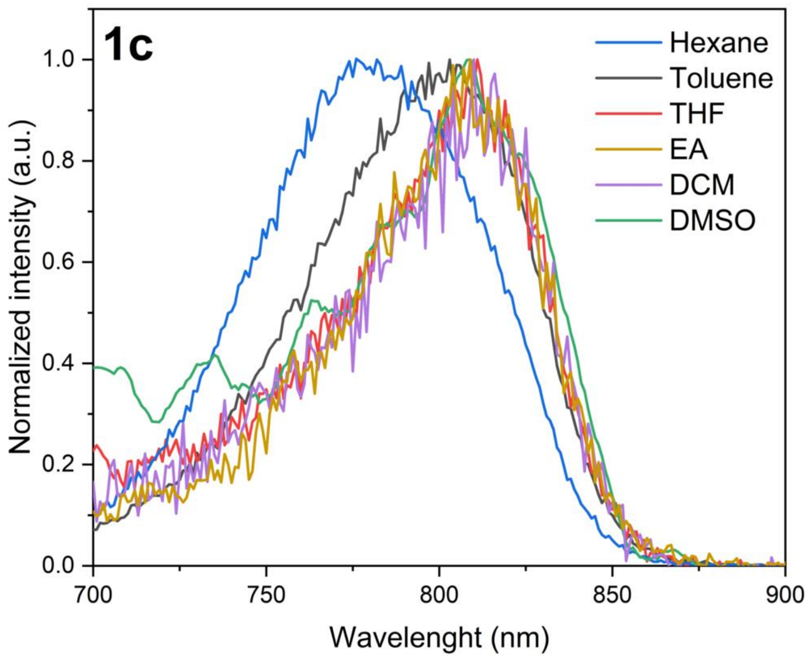

2. Absorption spectra of compounds 1a,b,c in different solvents

Figure S4 - Absorption spectra of compound **1a** in different solvents

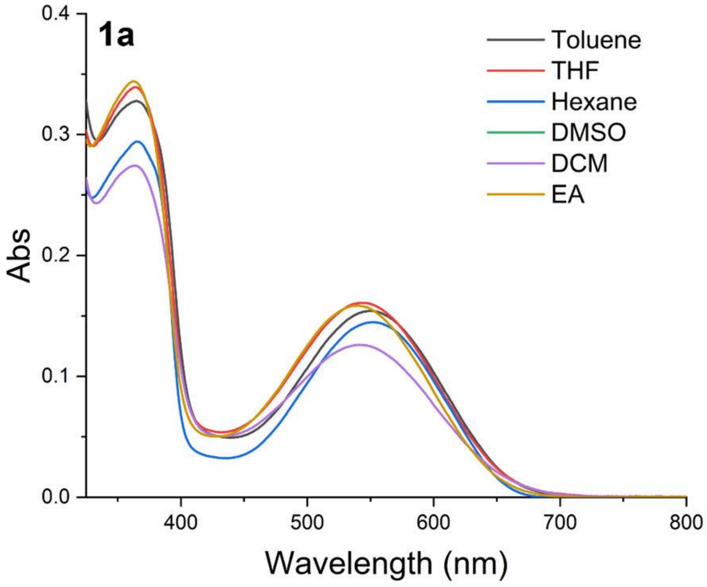

Figure S5 - Absorption spectra of compounds **1b** in different solvents

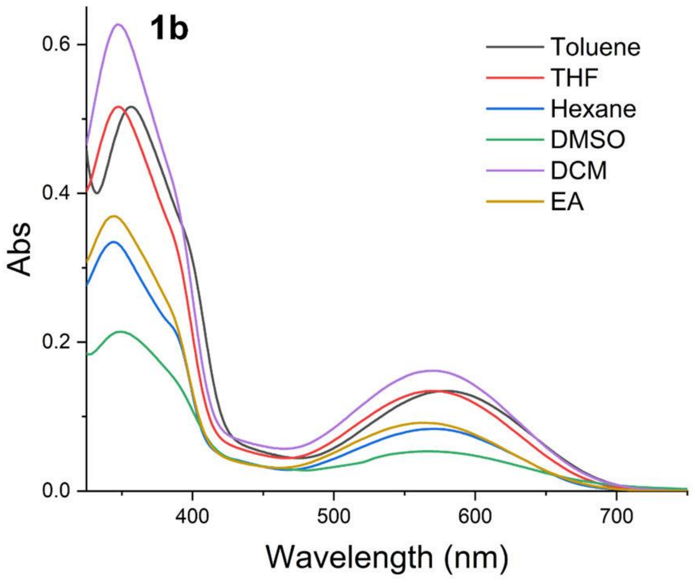

Figure S6 - Absorption spectra of compounds **1c** in different solvents

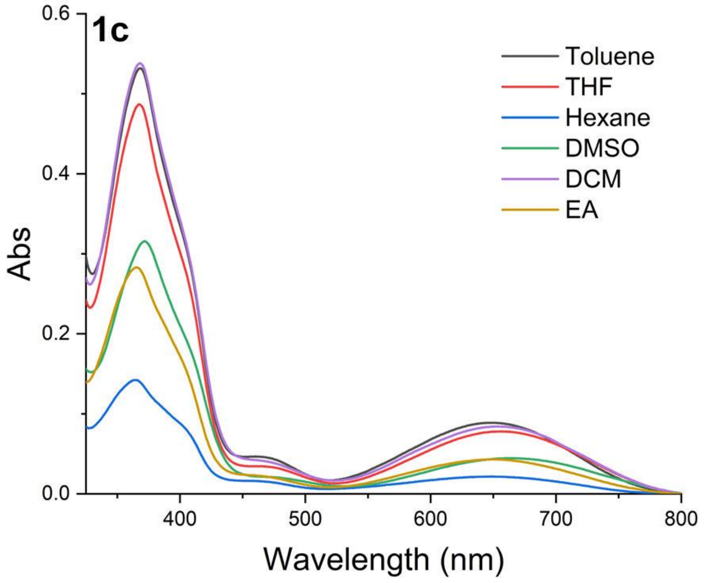

3. Fluorescence emission spectra of 1a,b,c in toluene at different concentrations

Figure S7 - Fluorescence emission spectra of **1a** in toluene at different concentrations (excitation at 365 nm)

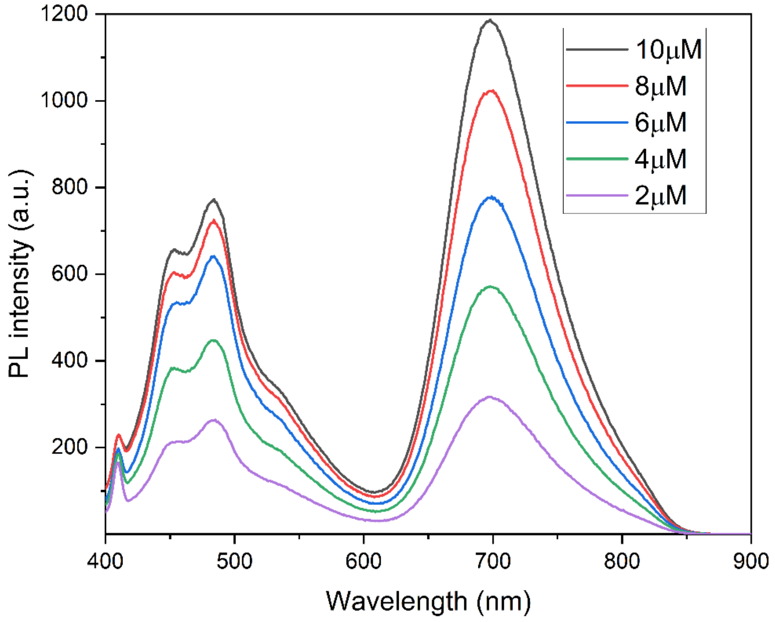

Figure S8 - Fluorescence emission spectra of **1b** in toluene at different concentrations (excitation at 347 nm)

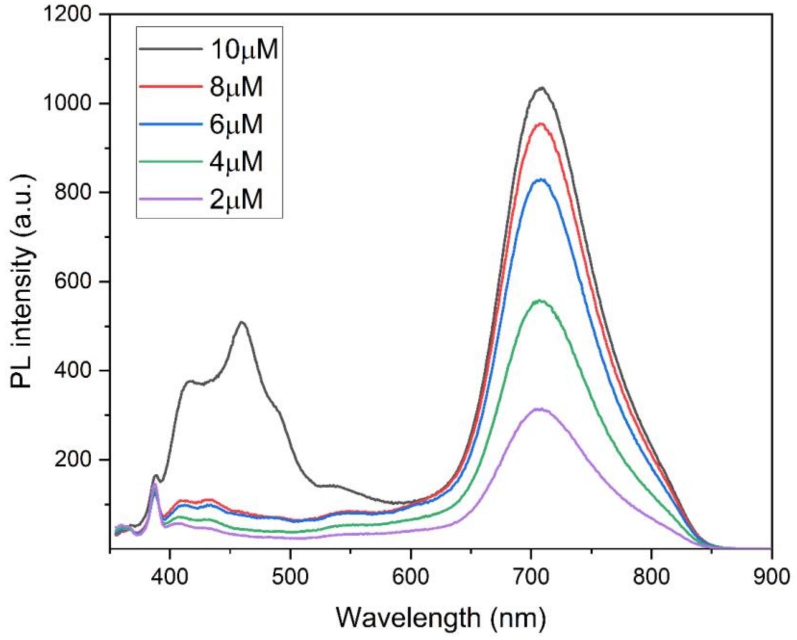

Figure S9 - Fluorescence emission spectra of **1c** in toluene at different concentrations (excitation at 368 nm)

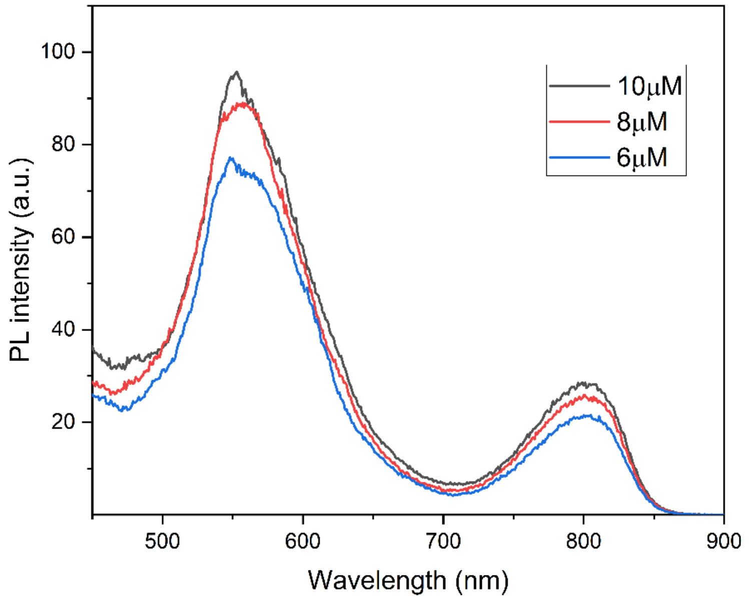

4. Absorption and fluorescence emission spectra of 1c in PMMA

Figure S10 - Absorption (red line) and fluorescence emission (black line) spectra of **1c** in PMMA (concentration 1.2 weight %) following excitation at 372 nm

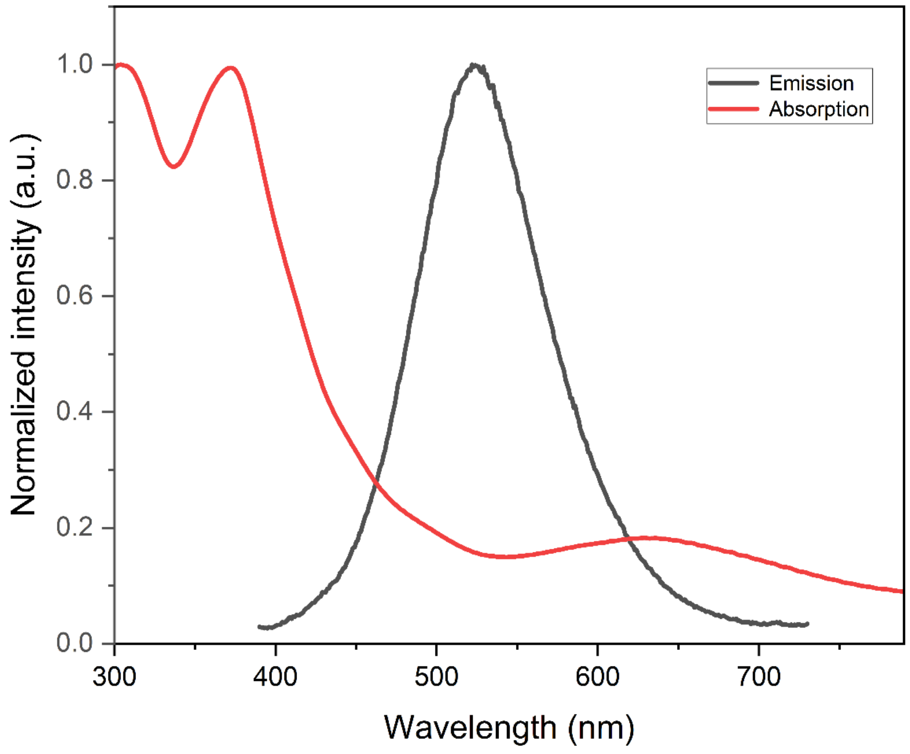

5. P(V) and I(V) curves of photovoltaic cell in the presence and absence of the LSC

Figure S11 - P(V) (blue lines) and I(V) (black lines) curves of the photovoltaic cell in the presence (solid line) and absence (dash line) of the best performing LSC (compound **1c**, 1.2% wt dispersion in PMMA).

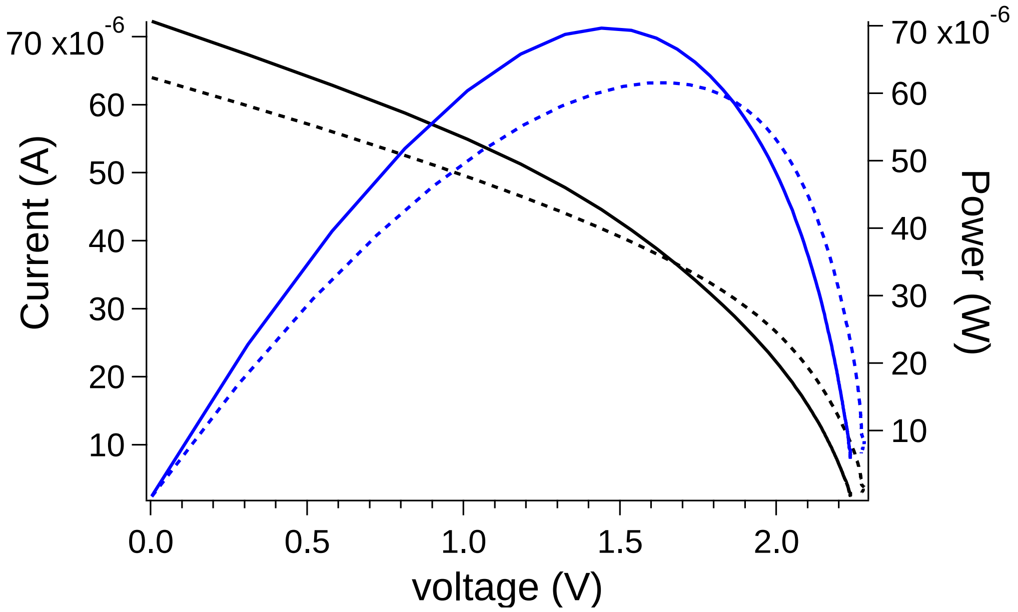

6. Copies of ^{1}H and ^{13}C NMR spectra of compounds **1a,b,c**

1a-1H_400MHz
XY16_f17-35

^{1}H NMR (400 MHz, THF) δ 8.46 (s, 2H), 8.18 – 8.11 (m, 4H), 7.30 – 7.25 (m, 8H), 7.15 – 7.11 (m, 8H), 7.11 – 7.08 (m, 4H), 7.06 – 7.01 (m, 4H).

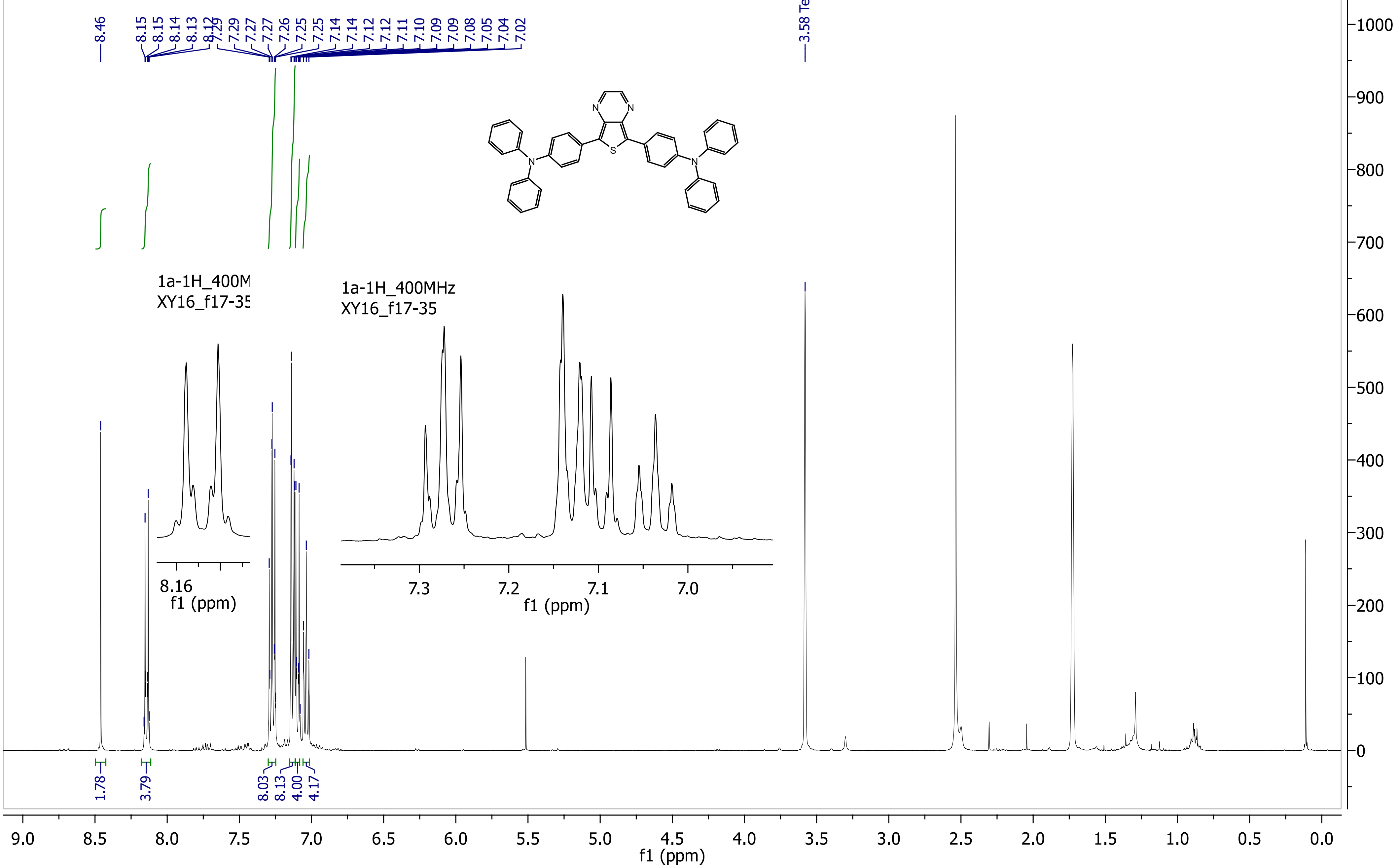

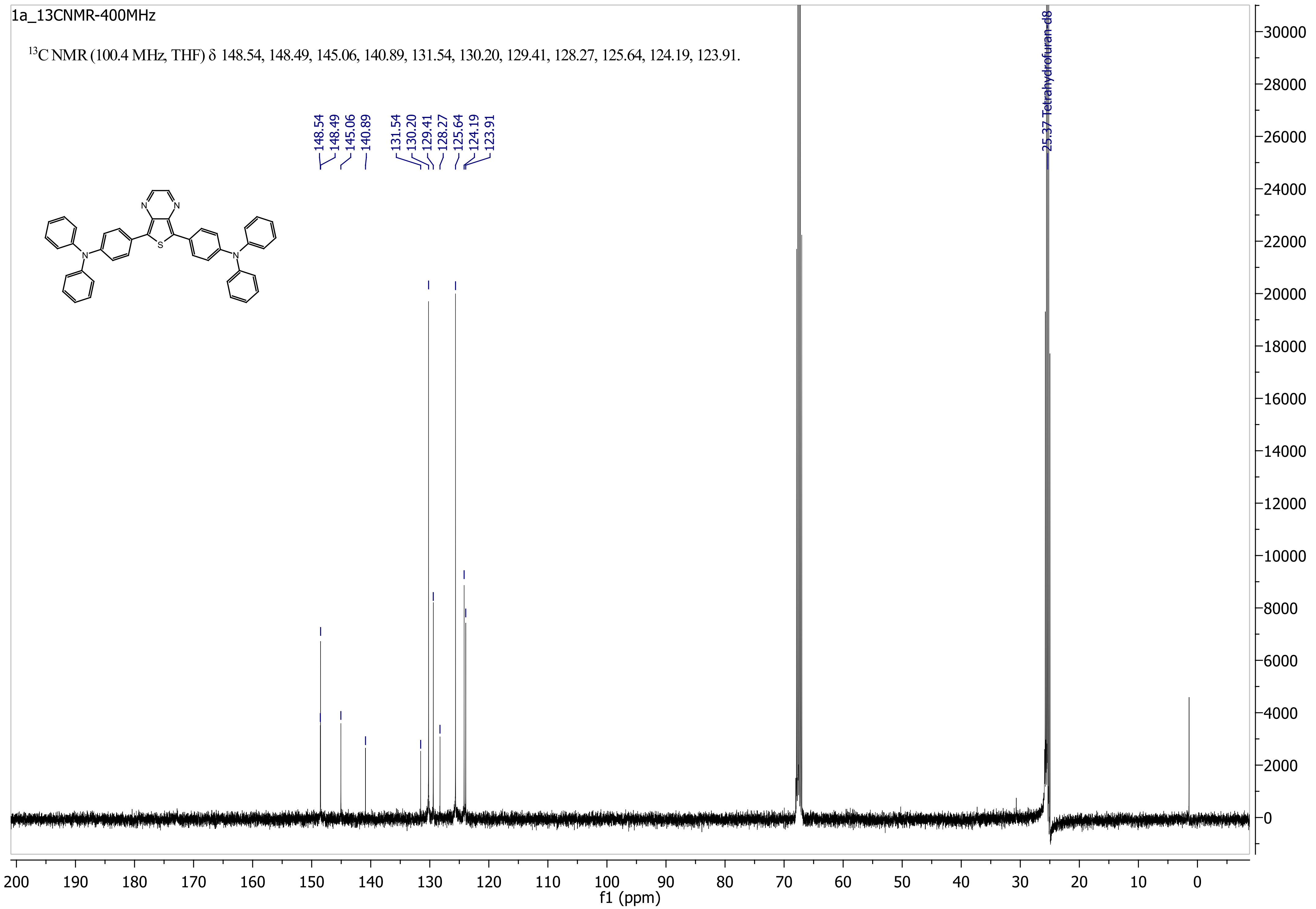

1a_13CNMR-400MHz
^{13}C NMR (100.4 MHz, THF) δ 148.54, 148.49, 145.06, 140.89, 131.54, 130.20, 129.41, 128.27, 125.64, 124.19, 123.91.
148.54
148.49
145.06
140.89
131.54
130.20
129.41
128.27
125.64
124.19
123.91
25.37 Tetrahydrofuran-d8
N
S
f1 (ppm)

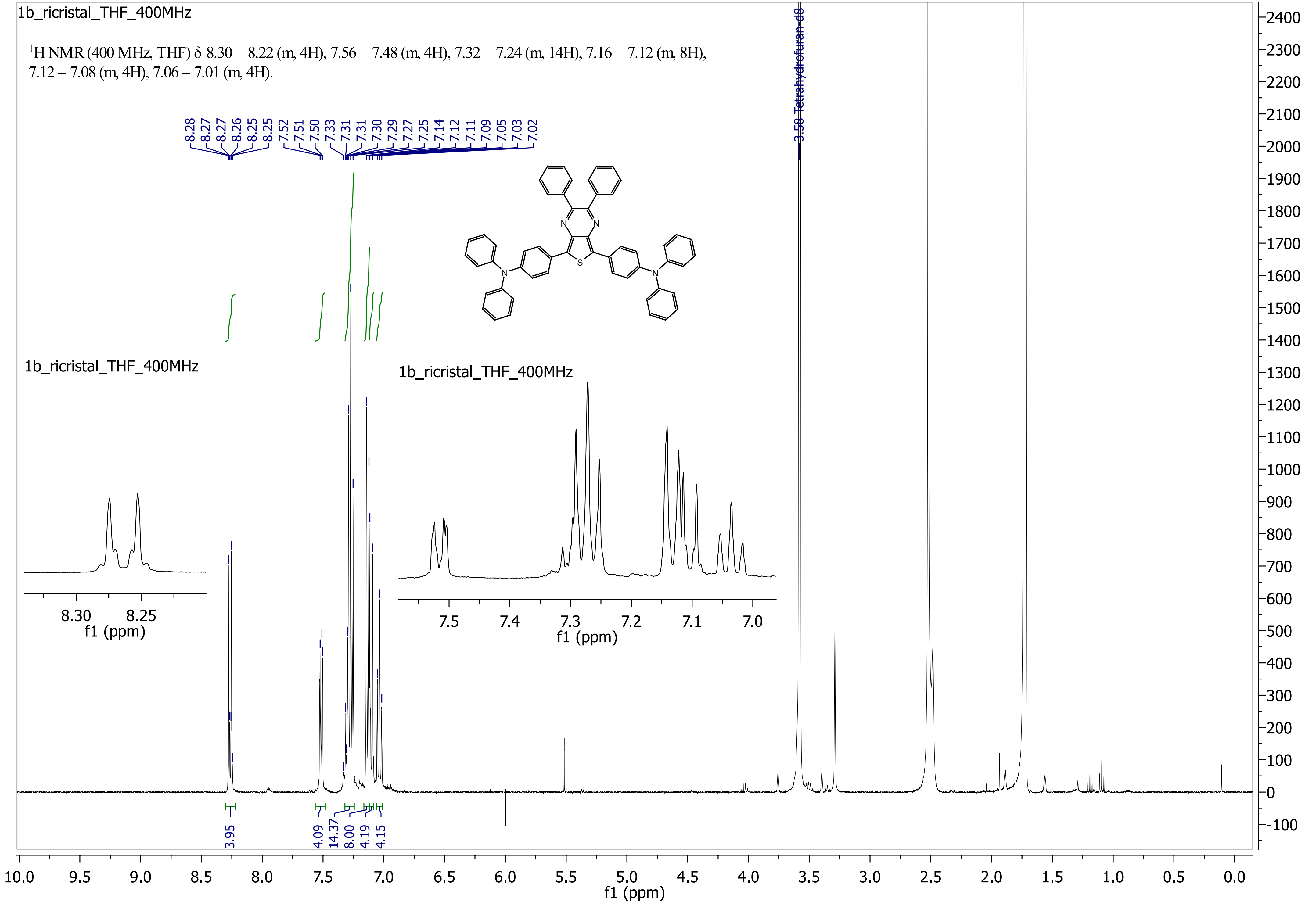
1b_ricristal_THF_400MHz
¹H NMR (400 MHz, THF) δ 8.30 – 8.22 (m, 4H), 7.56 – 7.48 (m, 4H), 7.32 – 7.24 (m, 14H), 7.16 – 7.12 (m, 8H), 7.12 – 7.08 (m, 4H), 7.06 – 7.01 (m, 4H).
8.28 8.27 8.27 8.26 8.25 8.25 7.52 7.51 7.50 7.33 7.31 7.31 7.30 7.29 7.27 7.25 7.14 7.12 7.11 7.09 7.05 7.03 7.02
3.58 Tetrahydrofuran-d8
N
S
3.95
4.09
14.37
8.00
4.19
4.15
1b_ricristal_THF_400MHz
8.30
8.25
f1 (ppm)
1b_ricristal_THF_400MHz
7.5
7.4
7.3
7.2
7.1
7.0
f1 (ppm)
10.0 9.5 9.0 8.5 8.0 7.5 7.0 6.5 6.0 5.5 5.0 4.5 4.0 3.5 3.0 2.5 2.0 1.5 1.0 0.5 0.0
f1 (ppm)
2400 2300 2200 2100 2000 1900 1800 1700 1600 1500 1400 1300 1200 1100 1000 900 800 700 600 500 400 300 200 100 0 -100

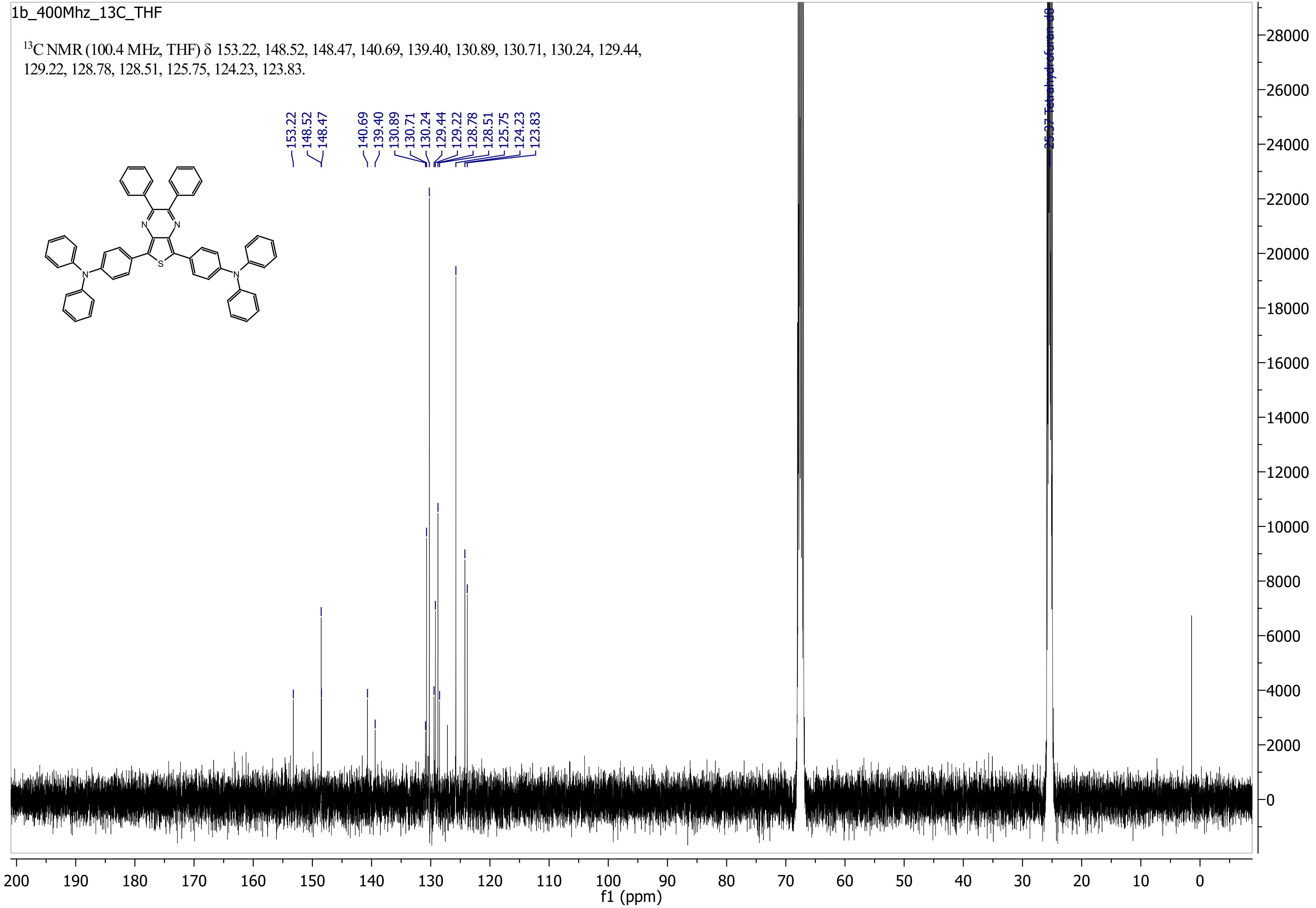
1b_400Mhz_13C_THF
^{13}C NMR (100.4 MHz, THF) δ 153.22, 148.52, 148.47, 140.69, 139.40, 130.89, 130.71, 130.24, 129.44, 129.22, 128.78, 128.51, 125.75, 124.23, 123.83.
153.22
148.52
148.47
140.69
139.40
130.89
130.71
130.24
129.44
129.22
128.78
128.51
125.75
124.23
123.83
25.37 Tetrahydrofuran-d8
N
S
f1 (ppm)

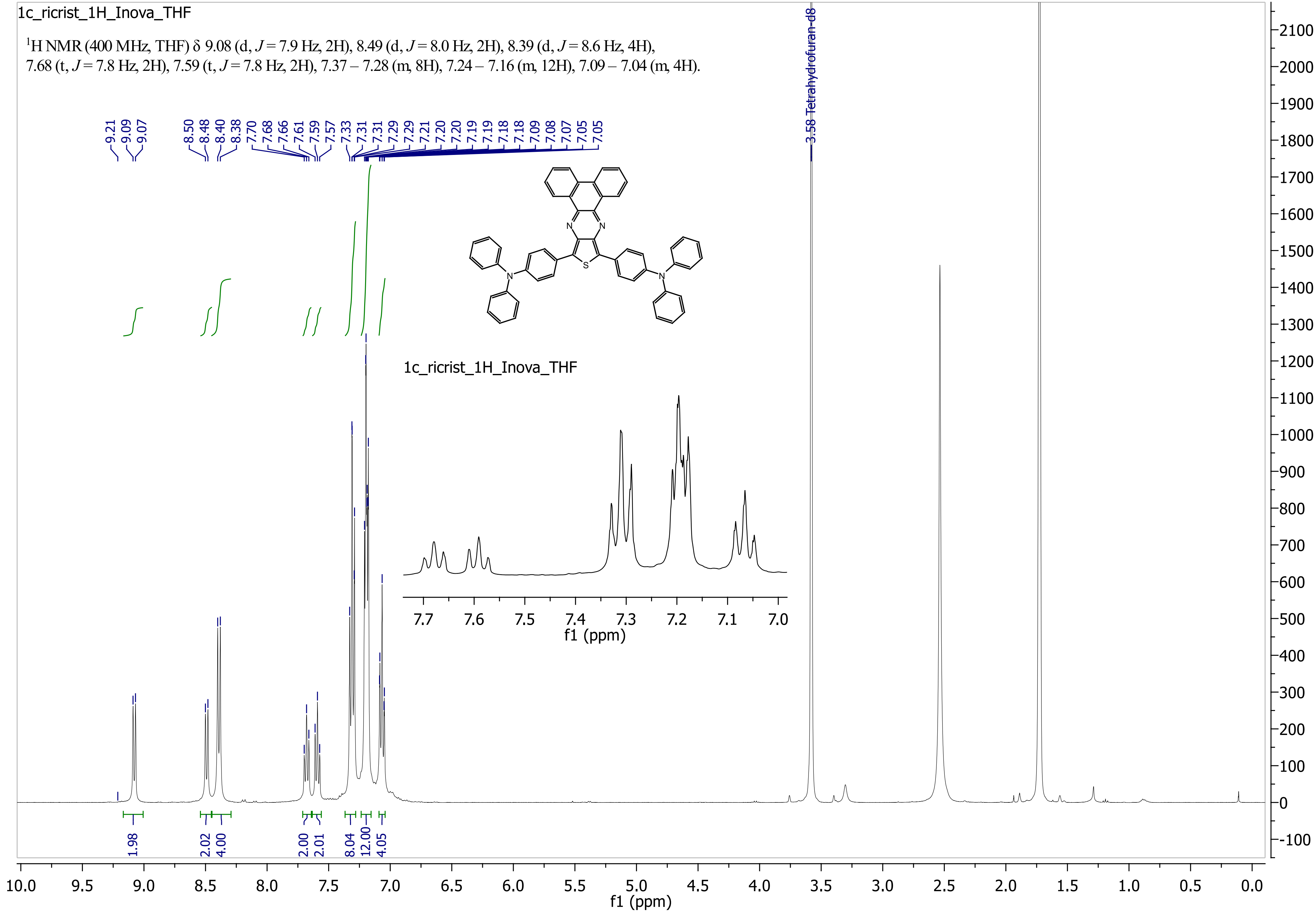
1c_ricrist_1H_Inova_THF
^{1}H NMR (400 MHz, THF) δ 9.08 (d, J = 7.9 Hz, 2H), 8.49 (d, J = 8.0 Hz, 2H), 8.39 (d, J = 8.6 Hz, 4H), 7.68 (t, J = 7.8 Hz, 2H), 7.59 (t, J = 7.8 Hz, 2H), 7.37 – 7.28 (m, 8H), 7.24 – 7.16 (m, 12H), 7.09 – 7.04 (m, 4H).
9.21 9.09 9.07 8.50 8.48 8.40 8.38 7.70 7.68 7.66 7.61 7.59 7.57 7.33 7.31 7.31 7.29 7.29 7.21 7.20 7.20 7.19 7.19 7.18 7.18 7.09 7.08 7.07 7.05 7.05
3.58 Tetrahydrofuran-d8
1c_ricrist_1H_Inova_THF
1.98
2.02
4.00
2.00
2.01
8.04
12.00
4.05
f1 (ppm)
f1 (ppm)

1c_13CNMR-400MHz

^{13}C NMR (100 MHz, THF) δ 149.90, 148.60, 148.46, 143.04, 140.44, 133.55, 131.84, 131.48, 130.53, 130.28, 129.31, 128.93, 127.22, 127.10, 125.79, 124.24, 124.07.

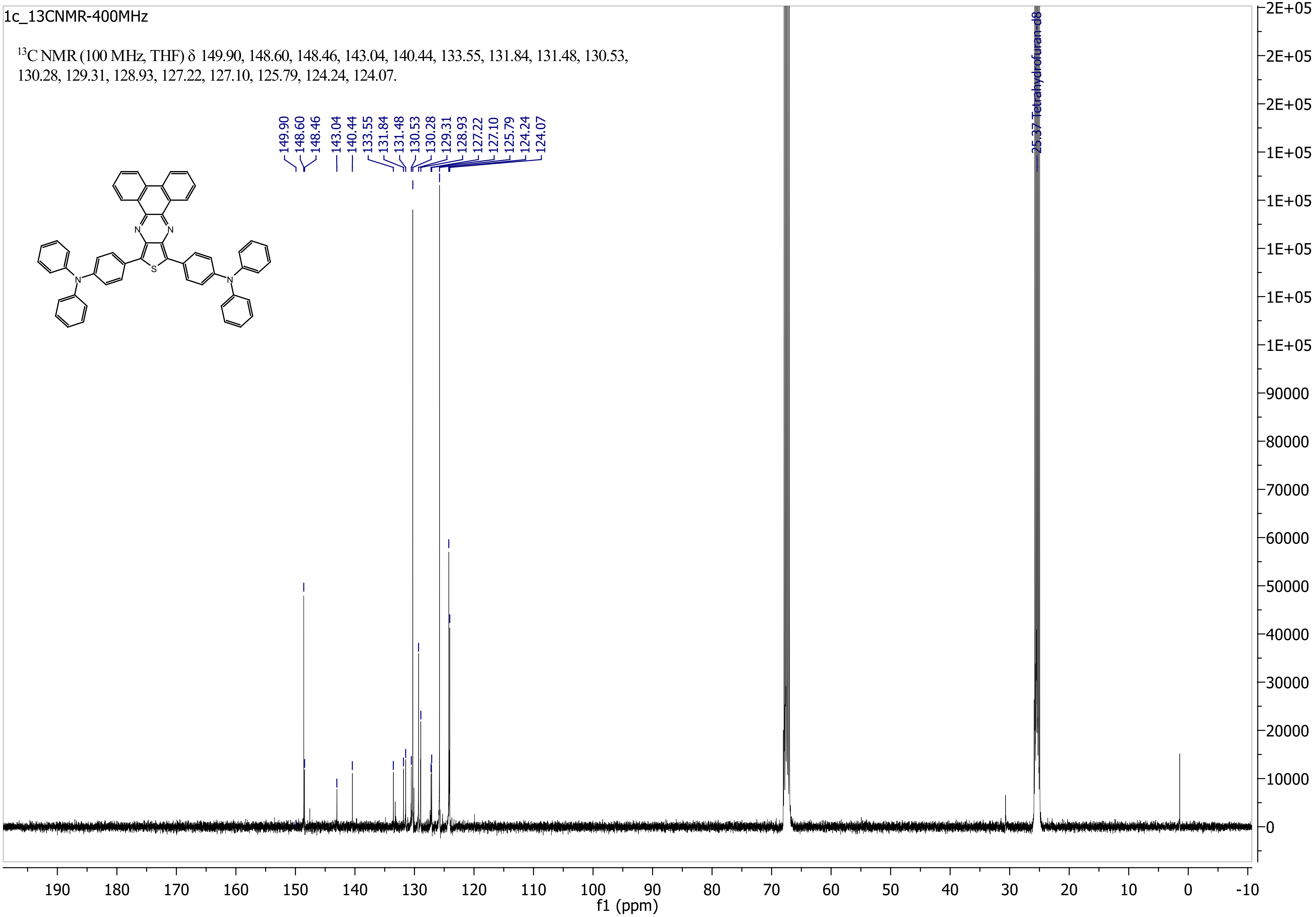

Supplement: Supplementary file 1 [file molecules-26-05428-s001.zip › molecules-1340477-supplementary.pdf]
